# Integrated use of finite element analysis and gaussian process regression in the structural analysis of AISI 316 stainless steel chimney systems

**DOI:** 10.1038/s41598-025-21678-z

**Published:** 2025-10-29

**Authors:** Kursat Tanriver, Ayhan Etyemez, Mustafa Ay

**Affiliations:** 1 Faculty of Engineering and Natural Sciences, Department of Mechatronics Engineering, Istanbul Health and Technology University, Beyoglu, Istanbul, 34445 Turkey; 2 Faculty of Technology, Department of Mechanical Engineering, Marmara University, Maltepe, Istanbul, 34854 Turkey

**Keywords:** Chimney system, FEM analysis, Gaussian process regression (GPR) stress prediction, Structural analysis, Engineering, Materials science, Mathematics and computing

## Abstract

This study aimed to conduct a comprehensive structural analysis and machine learning-assisted predictive modelling of a chimney system manufactured from 2 mm thick AISI 316 stainless steel with a diameter of Ø500 mm. The primary motivation of this work was to examine, in detail, the structural behavior of chimney modules under various force and pressure conditions using conventional methods, and to develop a reliable model capable of performing parametric predictions for new scenarios based on the acquired data. The scope of the study encompassed finite element analyses of both the entire chimney system and 3-meter-long intermediate modules, field tests, and the application of the Gaussian Process Regression (GPR) machine learning model. In the analysis of the entire chimney system under an applied force of 22,000 N, a maximum stress of 28 MPa and a safety factor of 8.39 were observed in the chimney clamps. The total deformation was found to be 0.58 mm, which is within acceptable limits. In the structural analysis of the intermediate chimney modules under a force of 1000 N and an internal pressure of 5 MPa, a maximum stress of 11,984 MPa, a safety factor of 1.71, and a total deformation of 0.46 mm were determined, all of which are consistent with the literature. The accuracy of these analyses was validated through pressure and leakage tests conducted in accordance with the EN 1859 standard. The developed GPR machine learning model demonstrated exceptionally high accuracy (R² > 0.999) in predicting Von Mises stress values, providing reliable forecasts with an error rate of less than 3% when compared to ANSYS simulation outputs. However, in predicting total deformation values, error rates exceeded 70%, indicating that the model was less sensitive in low-amplitude deformation cases. These findings suggest that the GPR model can generate reliable predictions for Von Mises stress a more critical parameter than total deformation in chimney design. By integrating conventional structural analysis methods with advanced machine learning techniques, this study demonstrates the potential of predictive modeling as an efficient and reliable tool in engineering design processes, making a significant contribution to the field’s body of knowledge.

## Introduction

 In modern industrial facilities, the efficient and safe discharge of exhaust gases is crucial for both process sustainability and employee health^1^. Particularly in high-rise plants and power stations, chimney systems are often positioned within shafts and designed in an integrated manner with the building structure^2^. The design of chimneys, including the materials used in their construction, connection details, the loads to which they are subjected, and the internal pressures they experience, should be evaluated through detailed engineering analyses.

In recent years, numerous experimental, numerical, and analytical studies in the literature have focused on strength, service life, performance optimization, and risk assessment of these structural components. Existing research has examined both the structural strength of steel and reinforced concrete chimney systems, as well as their behavior under various loading conditions. Gupta et al.^3^ modelled and analysed a reinforced concrete (RC) chimney structure using the finite element method under both wind loads and temperature variations, demonstrating that the structural integrity of the system was maintained. Similarly, Vatansever and Çayır^4^ analyzed the nonlinear dynamic behavior of steel chimneys and reported that, compared to earthquake loads, wind loads had a more decisive influence on top displacement. These studies indicate that, in chimney design, not only the magnitude but also the type of loads should be prioritized in the evaluation process.

Studies specifically addressing seismic effects have mainly focused on vibration mitigation techniques. Longarini et al.^5^ showed that the application of Tuned Mass Dampers (TMDs) in reinforced concrete chimneys enhances seismic resistance. At the same time, Hernández Barrios et al.^6^ evaluated the performance of TMD devices in reinforced concrete chimneys by considering soil–structure interaction using the Force Analogy Method (FAM) approach. In material-focused approaches, Tuhta and Günday^7^ demonstrated that the application of MgO nano-coatings on steel chimneys reduces both displacement and stress levels, indicating that surface materials indirectly contribute to damping characteristics. Similarly, Henao et al.^8^ reported that early damage due to stress-corrosion cracking in 316 l stainless steel chimneys is concentrated particularly in welded regions and characterized through detailed metallurgical analyses.

Vibration behavior and structural stability have been widely studied under wind load effects. Kumar^9^ optimized Guy Rope support systems to reduce lateral displacement, while Allaboudi and Ahmida^10^ investigated the influence of support ring positioning on vibration behavior using ANSYS-based harmonic analyses. Field-based studies, such as that of Gürsoy et al.^11^, emphasise that on-site measurement and analysis of existing chimneys are critical for identifying structural behaviors that cannot be fully captured through computer-based modelling alone.

From a fire safety perspective, Drozdzol et al.^12^ conducted a thermal analysis of perlite-based chimney and wooden ceiling junctions, revealing that even when the chimney temperature reached 38 °C, the wooden ceiling temperature remained within the safe limit of 28 °C, thus ensuring compliance with fire safety requirements. Additionally, Carvalho and da Silva^13^ highlighted the potential to improve energy efficiency by utilizing chimney gases in the drying process of ceramic bricks.

In comparative analyses based on standards and regulations, Paswan and Mishra^14^ evaluated the structural performance of steel chimneys in accordance with IS and EN standards. Comparative studies conducted under different climatic conditions have shown that design decisions are strongly influenced by geographical context. Investigating the causes of structural failures, Mukhopadhyay et al.^15^ demonstrated that welding defects and improper assembly techniques could lead to structural collapse during storms. In renewable energy applications, Jasim et al.^16^ and Behera et al.^17^ analyzed solar chimneys in terms of power generation and ventilation capacity, emphasising that hybrid systems possess significant potential for efficiency and sustainability.

Dong et al.^18^ investigated stress concentrations in reinforced concrete chimneys with openings under seismic loads through parametric analysis and the finite element method (FEM), evaluating the effects of wall thickness, opening size, and location. Furthermore, the study proposed reinforcement design recommendations around the openings, offering a method that can significantly reduce stress concentrations. Longarini et al.^5^ conducted a comprehensive investigation on enhancing the performance of seven reinforced concrete chimneys under environmental and seismic loads by retrofitting them with Tuned Mass Dampers (TMDs). The study evaluated the effects of TMDs on top displacement, base shear, base moment, and energy distribution. Furthermore, the dynamic behavior of the chimneys was modelled using the Finite Element Method (FEM), and the effectiveness of the study was numerically validated under different seismic scenarios.

Wu et al.^19^ analyzed the sealing performance of V-band clamps used in the exhaust systems of marine diesel engines. Their results indicated that thermal expansion effects under high temperature and pressure increased gasket contact pressure, enabling the safe application of higher preload values when optimized. This study provided valuable engineering input for the design of secure connection systems under high-temperature conditions.

Natarajan et al.^20^ experimentally and numerically investigated the influence of flow parameters on the performance of solar chimney systems with semi-elliptical collector geometry using ANSYS-FLUENT, revealing that models with fully elliptical curvature achieved the highest increases in velocity and temperature. As a solution to the low efficiency of solar chimney power plant (SCPP) systems, Mirzamohammad et al.^21^ proposed a hybrid structure integrated with a gas turbine, enabling continuous energy production both during the day and at night. By transmitting hot gases underground through embedded pipes, they observed increased air temperatures and power outputs, up to 554% higher than those resulting from solar radiation intensity. Mandal et al.^22^ analyzed the effects of parameters such as collector height, chimney diameter, and collector inlet velocity on energy output in solar chimney design using artificial neural networks (ANNs), showing that reducing chimney inlet diameter and collector height significantly increased power output.

In a multidisciplinary study, Georgiev and Bogoevska^23^ proposed a data-driven prognostic model for predicting and monitoring changes in continuously measured structural responses of an industrial steel chimney and a concrete arch dam. Based on the Polynomial Chaos Expansion approach, the models utilised environmental and response data collected from these structures, demonstrating considerable potential as a long-term monitoring tool for the autonomous assessment of structural behavior. Altıparmak and Akgün^24^ noted that with the development of industry, steel chimneys offer advantages over concrete chimneys, particularly in applications requiring high structures. They also evaluated steel chimney types, designs, project planning processes, manufacturing methods, and application areas in light of technological advancements. Güvel^25^ aimed to identify factors affecting slipform labor productivity in reinforced concrete chimney (RCC) construction and to develop a machine learning model to predict productivity. Based on 73 days of construction site data, the model revealed that variables such as daily rise height and formwork volume significantly influenced productivity, achieving a prediction accuracy of 90%.

Karahan and Balo^26^ designed an energy-efficient and low-emission chimney for a three-story building, taking into account the climate conditions of Bulanık, Muş, Türkiye. By incorporating stone wool insulation materials of varying thicknesses into the design, they analyzed performance. Evaluations performed using the Kesa Aladin simulation program indicated that a properly designed chimney can reduce emissions from natural gas boiler systems and enhance energy savings. Gleich^27^ reported on the construction of a 150-meter reinforced concrete industrial chimney for a thermal power plant, where an undesirable cold joint approximately 41 m above the base raised concerns about the structural safety of the chimney shell. Field inspections revealed significant structural damage despite the contractor’s contrary claims, leading to the assessment that the load-bearing capacity was inadequate and the recommendation that the newly built chimney be reinforced.

This study introduces a significant innovation in the field of structural analysis of chimney systems, particularly through the integration of machine learning approaches. It addresses a gap in the literature by successfully applying a Gaussian Process Regression (GPR) machine learning model to predict the structural parameters of chimney systems under various loading conditions. Comprehensive finite element analyses were performed using Solidworks Simulation and ANSYS Workbench, and the resulting data were employed to train the GPR model. This approach demonstrated the capability to predict stress and deformation values for loading scenarios not included in the initial dataset.

Notably, the GPR model achieved exceptionally high accuracy in predicting Von Mises stress, with an error rate of less than 3% and an R² value greater than 0.999, indicating its potential as a reliable predictive tool for this critical parameter in chimney design. Furthermore, the study not only relied on numerical analyses but also validated the accuracy of the structural assessments through field tests and leakage experiments conducted in accordance with the EN 1859 standard, thereby reinforcing its innovative and comprehensive methodology.

This integrated approach has the potential to accelerate the chimney design process and enable the development of more efficient and reliable designs in the future, offering a valuable contribution to the existing body of literature.

## Materials and methods

### Material

**Fig. 1:**
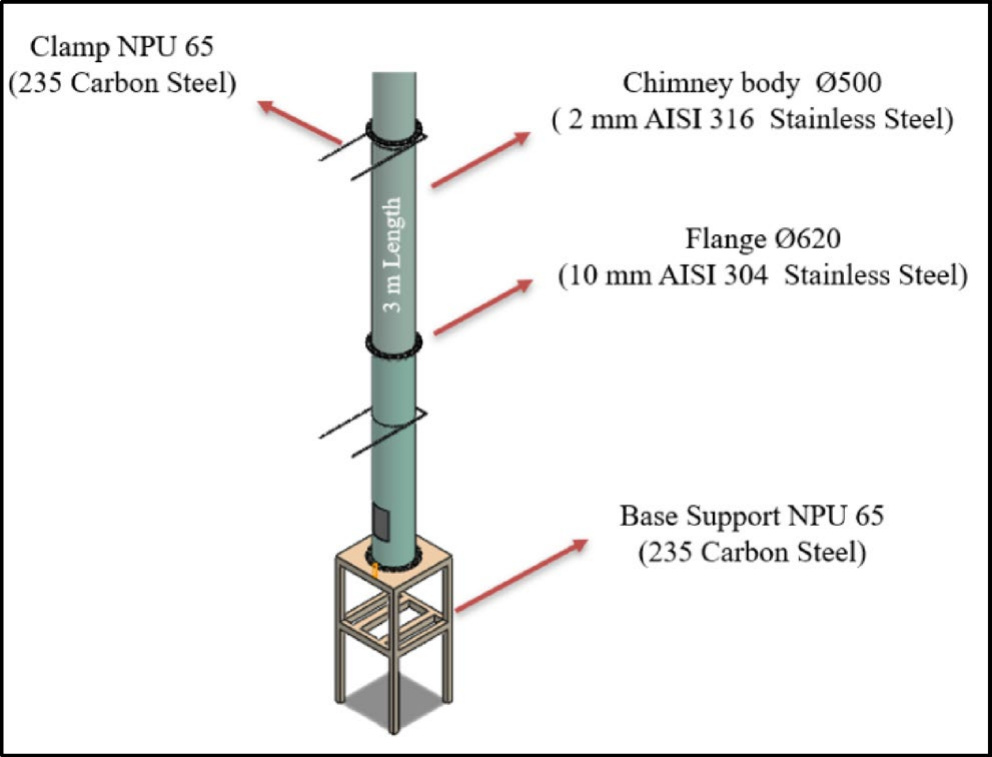
Chimney Design.

The chimney body was designed with a diameter of Ø500 mm and manufactured from 2 mm thick AISI 316 stainless steel. The chimney bodies were designed to be assembled in modules, each 3 m in length. For connecting these modules, flanged modules made of 10 mm thick AISI 304 stainless steel were added to both ends of each section. In addition, chimney clamps were designed to ensure the lateral stability of the system, and a base support was positioned at the bottom to bear the total weight of the chimney. These supports were designed using S235 material with NPU 65 profile sections. The chimney design is illustrated in Fig. 1.

The total weight of the chimney is approximately 2,236 kg. In calculating the chimney weight, the 10 cm stone wool insulation and the 0.6 mm aluminium cladding to be applied on the chimney were taken into account. The system weight is presented in Table 1.

**Table 1:**
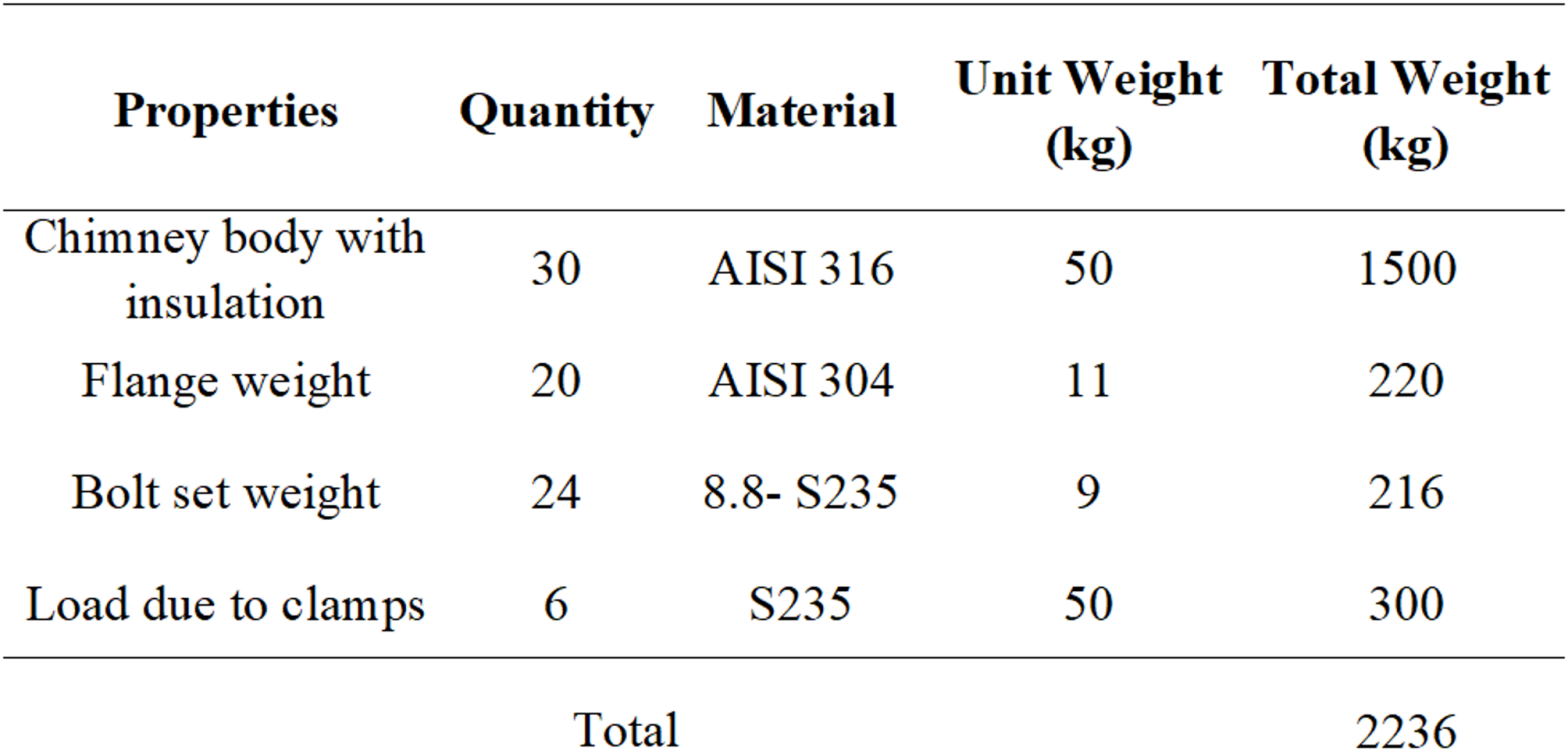
Chimney system weight Calculation.

### Method

In the finite element analysis of chimney systems, the structural analysis modules of software programs are generally employed^28^. In this manuscript, structural analyses were conducted for both the entire chimney system and the 3-meter intermediate modules.

Structural analyses were performed using SOLIDWORKS^®^ Simulation Student Edition 2024 SP5.0 [URL:https://www.solidworks.com/product/solidworks-simulation] and ANSYS^®^ Workbench Student 2024 R2 [URL:https://www.ansys.com/products/structures/ansys-mechanical].

SolidWorks Simulation was used to perform a global structural analysis of the complete chimney system under its own self-weight. Its direct integration with the CAD environment allowed for efficient evaluation of the overall stress distribution and deformation trends in the assembled structure, particularly at the clamps and base supports.

On the other hand, ANSYS Workbench was selected for the detailed analysis of the intermediate modules subjected to combined internal pressure and gravitational forces. The advanced meshing options, solver settings, and parametric analysis capabilities of ANSYS made it more suitable for generating a reliable dataset, which was subsequently used to train the Gaussian Process Regression (GPR) model.

Thus, the two software packages were applied in a complementary manner. SolidWorks Simulation was dedicated to capturing the holistic behavior of the whole chimney system, whereas ANSYS Workbench enabled refined analyses and parametric evaluations at the module level. Furthermore, the consistency observed between the results obtained from both platforms provided an implicit form of cross-verification, thereby enhancing the reliability and robustness of the numerical findings.

In Solidworks Simulation, structural analysis was conducted with the FFEPlus iterative solver. The chimney body and supporting components were modelled as solid meshes, with tetrahedral discretization. A mesh convergence study was carried out to ensure accuracy. In Solidworks, the final mesh consisted of 63,218 elements and 120,729 nodes, with an average element size of 74.5 mm. Element quality metrics showed less than 0.002% distorted elements, confirming adequate discretization. Boundary conditions were defined by assigning fixed supports to the base support beneath the entire chimney body and applying a load of 22,000 N in the negative z-direction at the center of gravity of the chimney body to represent its self-weight.

For the intermediate module analysis in ANSYS, structural analysis was conducted with the Sparse Direct Solver. The model was refined to 32,237 elements and 21,774 nodes, with an average element size of 10 mm. The minimum element quality was 0.09, and the maximum was 0.97. Tetrahedral discretisation was applied to the structural mesh. Boundary conditions were defined by assigning fixed supports to the bottom surface of the intermediate module, using a downward load of 1000 N in the negative z-direction at its center of gravity, and specifying an internal pressure of 5 MPa.

Validation was performed experimentally in accordance with the EN 1859 standard^29^, through pressure and leakage tests. The chimney modules were pressurized to 0.1 bar (twice the standard test pressure). The leakage test confirmed compliance with EN 1859 tolerances with a leakage rate less than 0.006 l/s/m².

Subsequently, a structural parametric analysis was performed, and the Gaussian Process Regression (GPR) model^30^ was applied to a dataset of 50 samples to predict experimental conditions not included in the original dataset.

## Results and discussion

### Full system FEM analysis

The total weight of 2,236 kg, as shown in Table 1, is effectively divided between two intermediate supports, resulting in a load of 1,118 kg on each support. However, to simulate a critical scenario, the static analysis was performed as if no intermediate support existed apart from the base support, by applying a load of 2,200 kg to the base support. The chimney is subjected to a compressive force of approximately W = 22,000 N (2,236 kg) due to its self-weight, as defined in the SolidWorks Simulation academic module.

Boundary conditions were assigned by applying fixed support at the base support beneath the chimney and at the chimney clamps. As the chimney installation remains within the shaft area and a compensator is used to account for potential vibration and thermal expansion, wind load and vibration effects were neglected in the calculations. The structural analysis was carried out considering only the load resulting from the system’s self-weight.

**Fig. 2:**
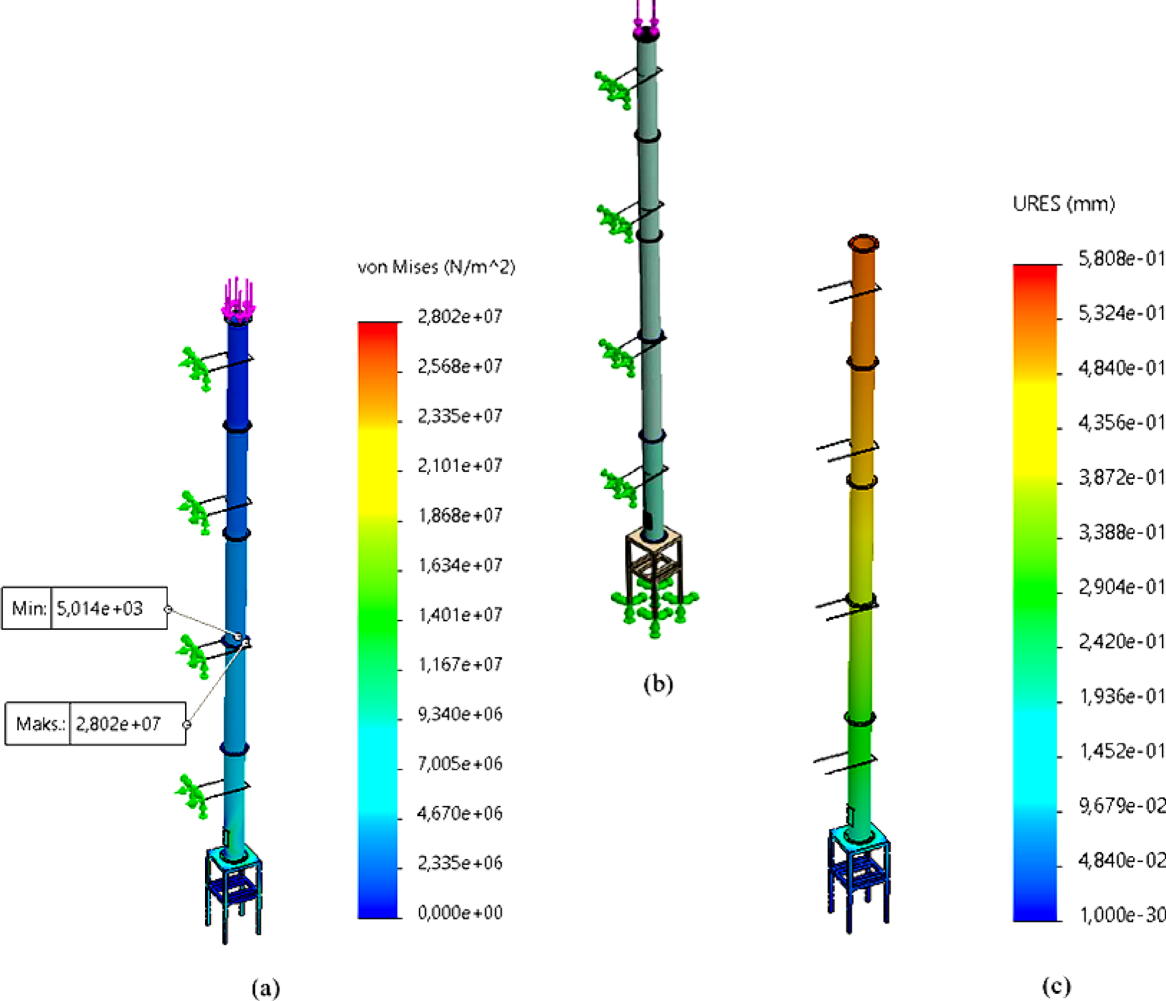
Structural analysis: **a**) Von Mises, **b**)3D Design, **C**) Total Deformation.

In Solidworks Analysis, the final mesh consisted of 63,218 elements and 120,729 nodes, with an average element size of 74.5 mm. The chimney body and supporting components were modeled as solid meshes, with tetrahedral discretization. A mesh convergence study was carried out to ensure accuracy. In Solidworks Simulation, structural analysis was conducted with the FFEPlus iterative solver. The finite element solution was iterated until the results became mesh-independent, and the final output is presented in Fig. 2.

According to these results, the maximum stress was found to be 28 MPa, occurring at the chimney clamps. Since the chimney clamps are made of S235 material, with a yield strength of 240 MPa^31^, the safety factor is calculated as 8.39. Furthermore, the total deformation was determined to be 0.58 mm, which is within acceptable limits for the chimney design.

### Part system FEM analysis

**Fig. 3:**
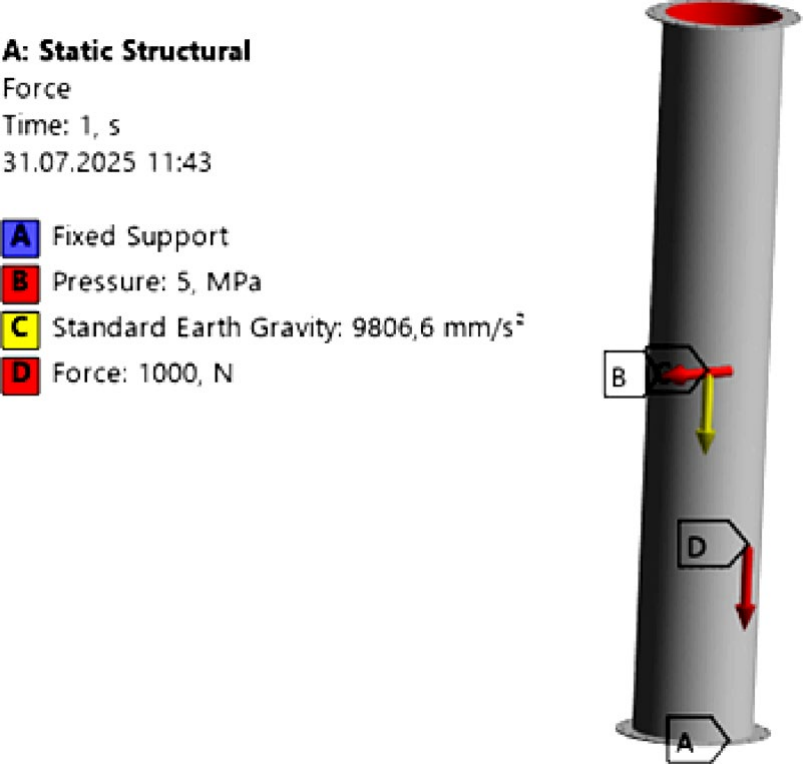
Boundary Conditions.

Here, the structural analysis was conducted by considering the 3-meter-long intermediate modules, manufactured from 2 mm-thick AISI 316 material, within the entire chimney system. Various boundary conditions and load cases were defined for the structural analysis of these intermediate modules. The boundary conditions are illustrated in Fig. 3.

**Fig. 4:**
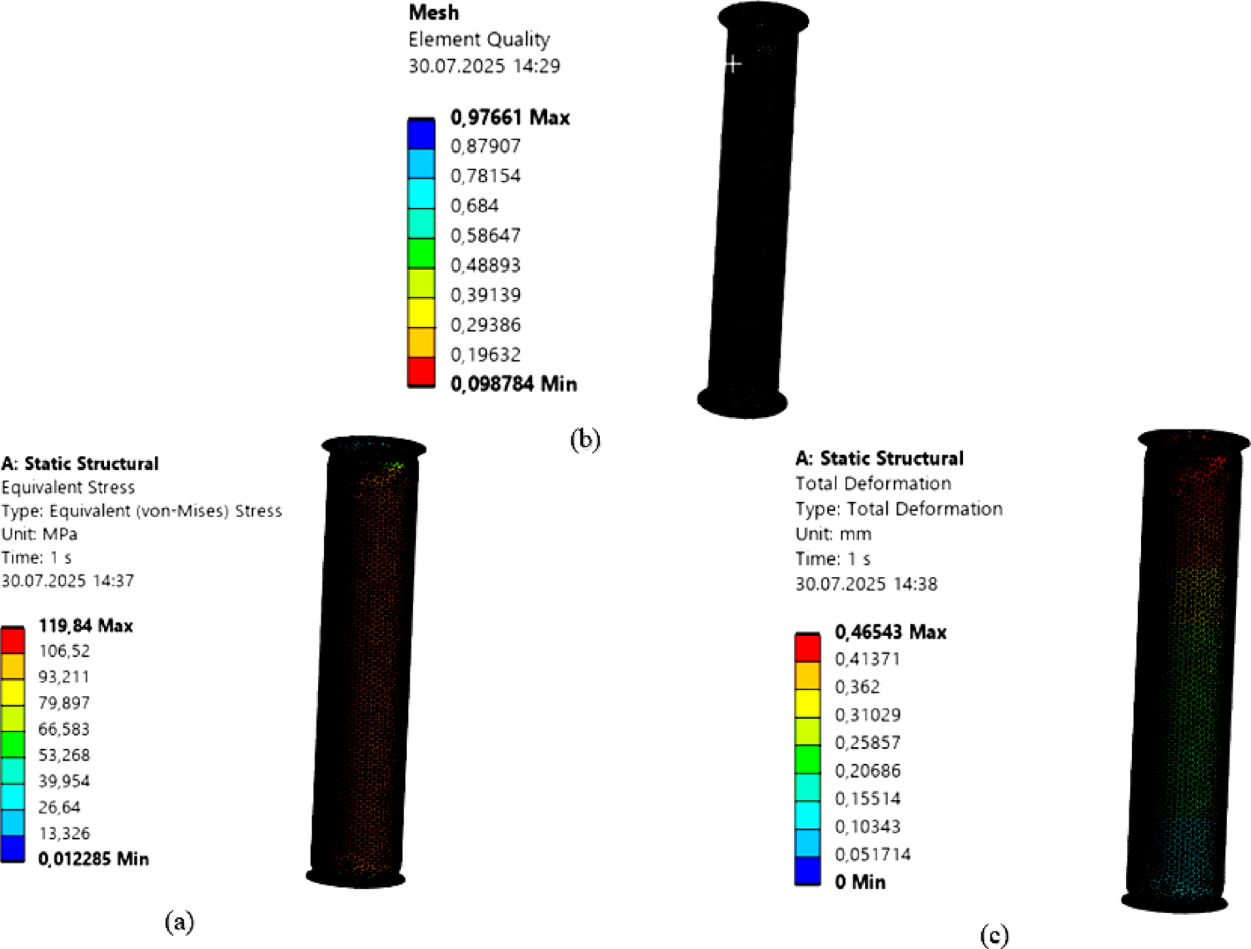
Structural Analysis **a**) Von-mises **b**) Element Quality **c**) Total deformation.

For this intermediate chimney module, a force of 1000 N, corresponding to its share of the total chimney system weight, was applied, along with an internal pressure of 5 MPa on its inner surface and the gravitational acceleration. In this way, the behavior of the pressurized module under its own weight and the applied load was calculated and examined. In this analysis, a mesh study was conducted in the ANSYS Workbench academic structural module. Structural analysis was performed with the Sparse Direct Solver. The model was refined to 32,237 elements and 21,774 nodes, with an average element size of 10 mm. The minimum element quality was 0.09, and the maximum was 0.97. Tetrahedral discretization was applied to the structural mesh. The Von Mises stress distribution is shown in Fig. 4. a, the mesh structure in Fig. 4.b, and the total deformation in Fig. 4.c.

In this case, the element quality was found to range from a minimum of 0.09 to a maximum of 0.97. Based on this mesh analysis, the maximum stress was determined to be 119.84 MPa, while the maximum total deformation was 0.46 mm.

Since the yield strength of the AISI 316 material used for the intermediate chimney module is at least 205 MPa^32^, the safety factor is calculated as 1.71. This value is consistent with those reported in the literature^33^. The total deformation of 0.46 mm is within acceptable limits for the chimney design.

### Experimental test

The 3-meter intermediate modules were connected using flanges to create the experimental setup. A 2 mm thick wire-reinforced graphite gasket was placed between the two flanges to ensure sealing. For joining the flanges, M20 × 60 bolts of grade 8.8 were used.

**Fig. 5:**
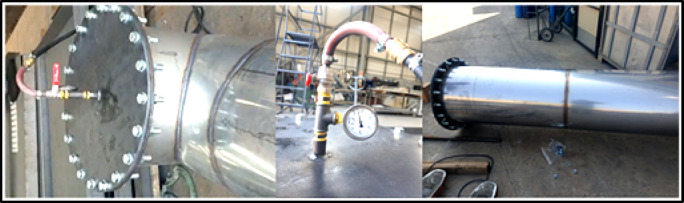
Pressure Test.

In this experimental setup, the open flanges of the connected modules were sealed with blind flanges, leaving a ½-inch coupling on one end for air injection. An air compressor was connected to the system for pressurization, and a manometer was installed to measure the pressure. The experimental setup is shown in Fig. 5.

Although the test pressure was required to be 5,000 Pa (0.05 bar), the system was pressurized to at least twice this value, 10,000 Pa (0.1 bar), to determine whether any deformation would occur in the chimney body sheet or welds. Following the test, visual inspection of the chimney assembly and body revealed no deformation, indicating consistency with the structural analysis results.

**Fig. 6:**
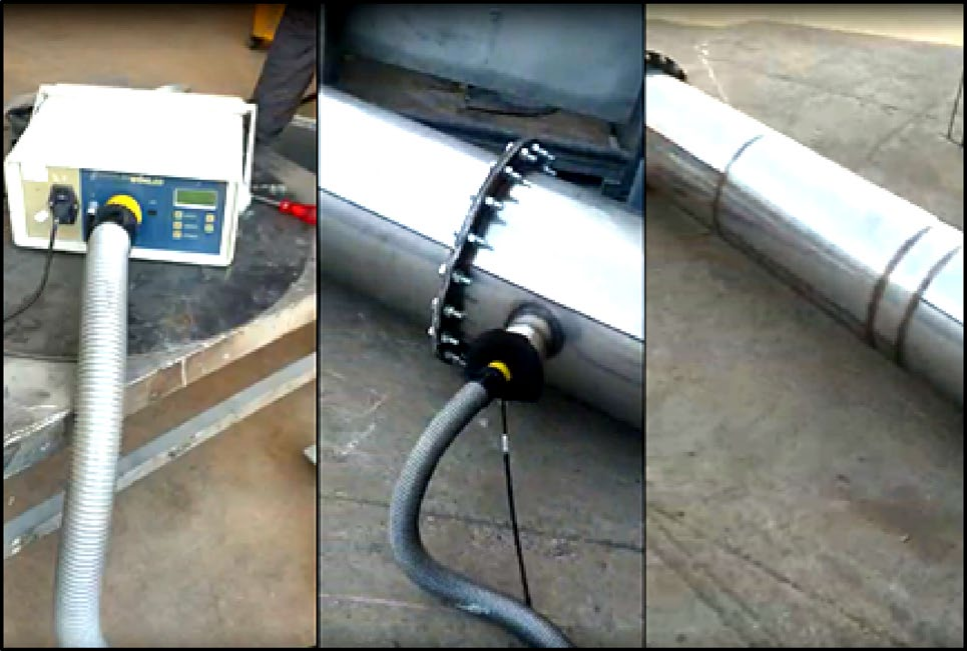
Leakage Test.

In addition, after the pressure test, a leakage test was conducted in accordance with the EN 1859 standard by preparing the experimental setup accordingly. A WOHLER DP 600 leakage testing device, compliant with this standard, was used in the experiments. Images from the leakage tests are presented in Fig. 6.

The leakage test results were found to be within the tolerances specified by the standards, and the test outcome was positive.

**Fig. 7:**
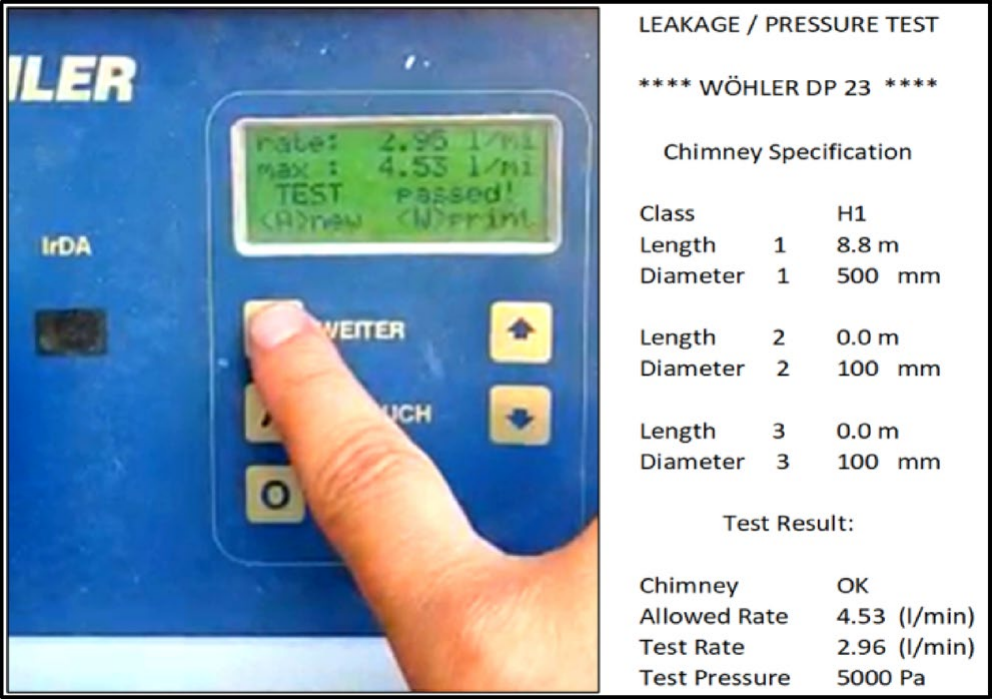
Leakage Test Result.

The leakage test confirmed compliance with EN 1859 tolerances with a leakage rate less than 0.006 l/s/m². This confirmed that the bolt connections, flange thicknesses, and gaskets were appropriately selected. The leakage test result is shown in Fig. 7.

## Machine learning

In this study, the Gaussian Process Regression (GPR) method, a statistical-based machine learning approach, was employed to process the datasets obtained from the structural analysis results generated using the ANSYS academic version.

### Parametric analysis

In the GPR method, 50 results were generated at randomly varying pressures and loads to serve as the dataset.

To obtain these results more efficiently, rather than individually, the parametric analysis feature of the ANSYS Academic Structural Analysis module was utilized.

The input variables of the dataset were defined as the applied pressure (P_1_) and the Z-directional force (P_2_). In contrast, the target variables were selected as the maximum total deformation (P_3_) and the maximum equivalent (Von Mises) stress (P_4_).

The program was then executed to generate a dataset of 50 samples. The parametric analysis dataset is summarized in Table 2.

**Table 2:**
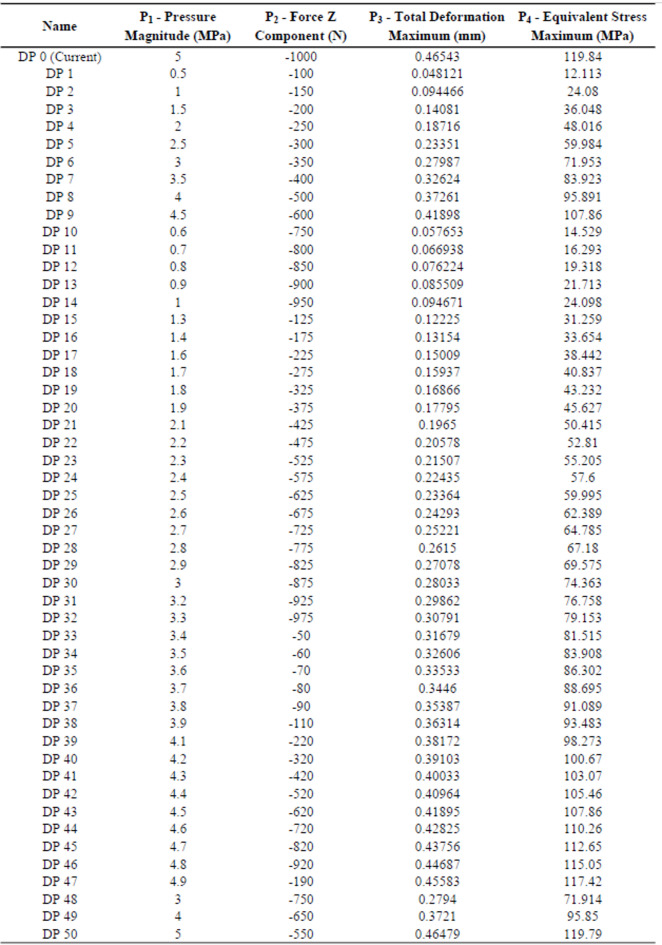
Parametric analysis data Set.

### Gaussian process regression (GPR)

In regression problems, the GPR method, which provides high accuracy, adopts a probabilistic approach to identify patterns within the dataset. This characteristic enables not only the prediction itself but also the assessment of the reliability of that prediction in engineering problems^34^.

GPR is based on a Gaussian process, defined as an infinite-dimensional joint distribution of random variables. The fundamental assumption is that a normal distribution can represent the model output for any input point, and that all points jointly follow a Gaussian distribution^35^.

A regression problem can be defined as follows^36^.

1 $$\:\text{y}=f\left(\text{X}\right)+\epsilon\:$$

Where:

$\:\text{X}\in\:{\mathbb{R}}^{n\times\:d}$ : Input matrix (nnn samples, ddd features).

$\:\text{y}\in\:{\mathbb{R}}^{n}$ : Observed output vector.

$\:f(\cdot\:)$ : Function to be learned.

$\:\epsilon\:\sim\:\mathcal{N}\left(0,{\sigma\:}_{n}^{2}\right)$ : Independently and identically distributed Gaussian noise.

GPR assumes that the function $\:f(\cdot\:)$ It is modeled by a Gaussian process, which is expressed in the following Eq. 

2 $$\:f\left(\mathbf{x}\right)\sim\:\mathcal{G}\mathcal{P}\left(m\left(\mathbf{x}\right),k\left(\mathbf{x},{\mathbf{x}}^{{\prime\:}}\right)\right)$$

Where;

$\:m\left(\mathbf{x}\right)$ : The mean function is generally taken to be zero, $\:m\left(\mathbf{x}\right)=0$)

$\:k\left(\mathbf{x},{\mathbf{x}}^{{\prime\:}}\right)$ : The most used kernel function, the Squared Exponential (RBF).

The covariance (kernel) function is expressed as follows.

3 $$\:k\left(\mathbf{x},{\mathbf{x}}^{{\prime\:}}\right)={\sigma\:}_{f}^{2}\text{e}\text{x}\text{p}\left(-\frac{1}{2{\mathcal{l}}^{2}}{\parallel\mathbf{x}-{\mathbf{x}}^{{\prime\:}}\parallel}^{2}\right)$$

Where:

$\mathcal{l}$ : Characteristic length scale.

$${\sigma\:}_{f}^{2}$$ : Signal variance.

Let the $\:\mathbf{y}$ be observed for the inputs $\:\mathbf{X}$ In the dataset. For a new input point $\:{\mathbf{x}}_{\text{z\:}}$The output prediction is expressed as a normal distribution, as given in the following Eq. 

4 $$\:{f}_{\text{*}}\mid\:\mathbf{X},\mathbf{y},{\mathbf{x}}_{\text{*}}\sim\:\mathcal{N}\left({\mu\:}_{\text{*}},{\sigma\:}_{\text{*}}^{2}\right)$$

5 $$\:\begin{array}{c}\\\:{\mu\:}_{\text{*}}={\mathbf{k}}_{\text{*}}^{\text{top}}{\left(K+{\sigma\:}_{n}^{2}I\right)}^{-1}\mathbf{y}\\\:\end{array}$$

6 $$\:{\sigma\:}_{*}^{2}=k\left({\mathbf{x}}_{*},{\mathbf{x}}_{{*}}\right)-{\mathbf{k}}_{\text{*}}^{\text{top}}{\left(K+{\sigma\:}_{n}^{2}I\right)}^{-1}{\mathbf{k}}_{*}$$

Where;

$\:K$ : Kernel matrix of the training data, $\:{K}_{ij}=k\left({\mathbf{x}}_{i},{\mathbf{x}}_{j}\right)$

$\:{\mathbf{k}}_{z}$ : Covariance vector between the new point and the training data

$\:{\mu\:}_{\text{z}}$ : Predicted meaning (model output)

$\:{\sigma\:}_{\text{*}}^{2}$ : Predicted variance (uncertainty)

Through this formulation, it is possible to compute not only the prediction but also the confidence intervals. This capability makes the GPR model highly valuable in engineering applications for decision support systems, reliability analyses, and uncertainty propagation.

**Fig. 8:**
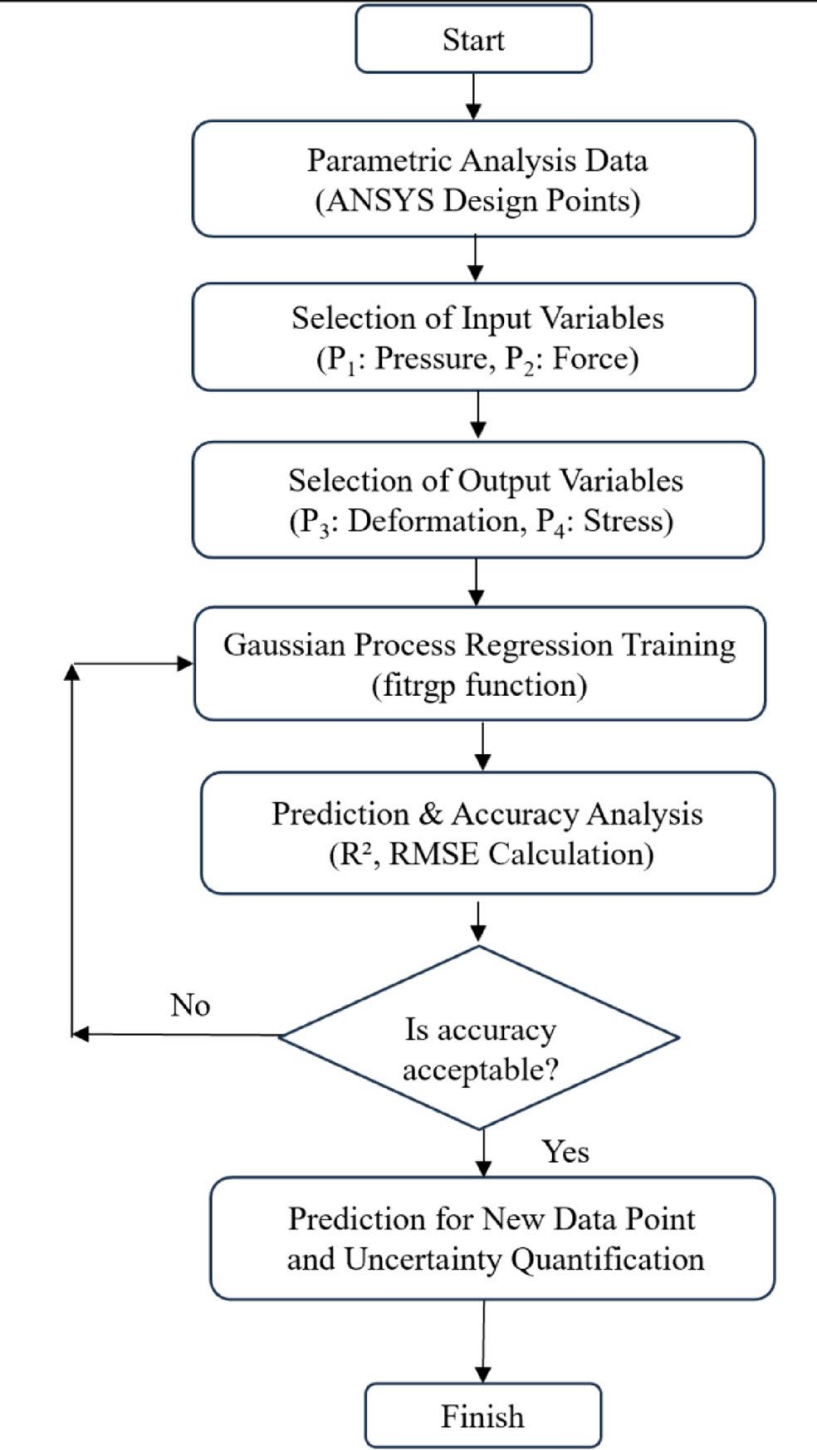
The GPR-based flowchart.

In this study, the GPR model was developed in MATLAB using 50 parametric analysis results obtained from ANSYS. The applied pressure (P1) and Z-directional force (P2) were used as the input variables, while the total deformation (P3) and equivalent (Von Mises) stress (P4) were selected as the target variables. The model was trained in MATLAB using the *fitrgp* function, and various kernel functions were tested to determine the most suitable configuration. The GPR flowchart is presented in Fig. 8.

**Fig. 9:**
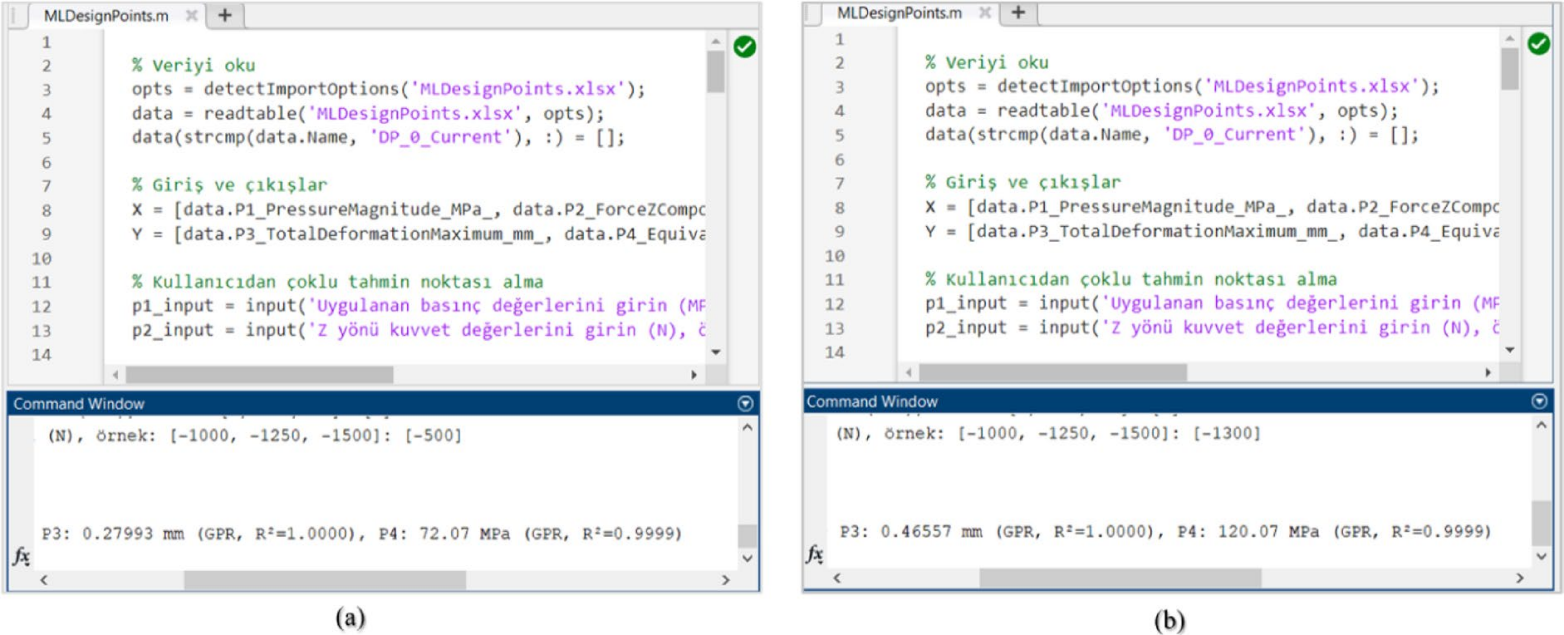
The GPR MATLAB Result Screen.

The GPR algorithm developed in MATLAB was executed with two additional external inputs that were not included in the initial dataset to predict the corresponding results. When the algorithm is run, it prompts the user to input new values. In this case, the algorithm was executed with input values of^3,5^ (MPa) and [−500, −1300] (N), yielding the corresponding results. The MATLAB output screen of the GPR algorithm is shown in Fig. 9.

First, the algorithm was executed using an input of 3 MPa pressure and − 500 N load. According to the results, the predicted Von Mises stress was 72.07 MPa, and the total deformation was 0.27 mm. Second, the algorithm was run with an input of 5 MPa pressure and − 1300 N load, yielding a predicted Von Mises stress of 120.07 MPa and a total deformation of 0.46 mm.

Based on these results, the GPR model achieved exceptionally high accuracy in predicting both deformation and stress values, with an R² value greater than 0.999, consistent with the literature^37,38^. This demonstrates that the model is highly suitable for producing rapid and reliable predictions, particularly when based on numerical results obtained from finite element analyses.

**Fig. 10:**
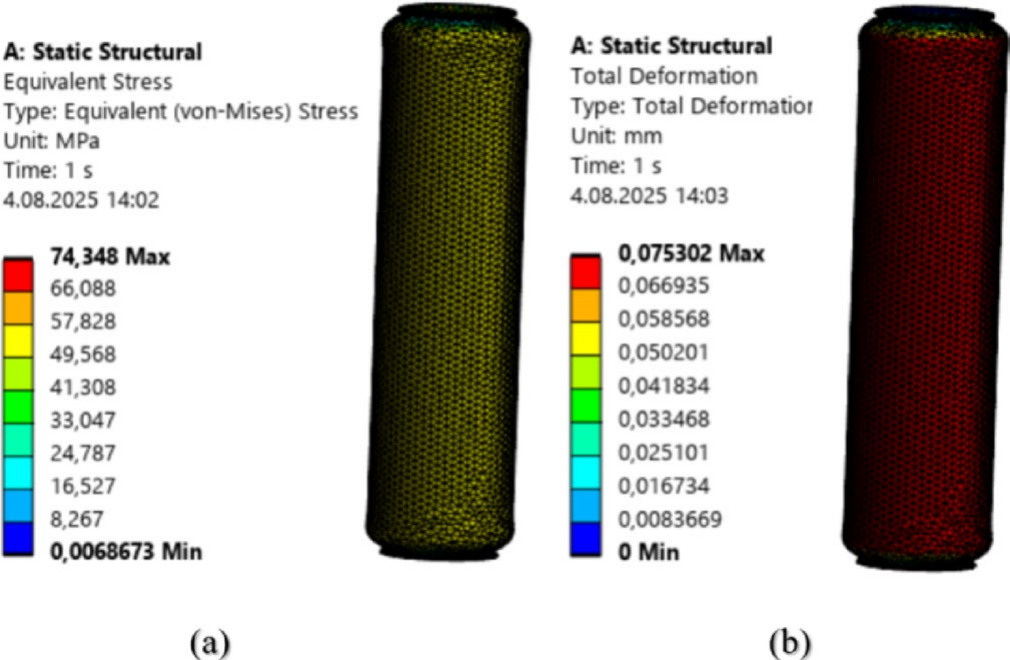
Structural Analysis **a**) Von Mises **b**) Total Deformation.

To compare the points predicted by the GPR algorithm with the structural analysis results from the ANSYS academic module, structural analyses were performed for the same input values. The structural analysis results for an input pressure of 3 MPa and a load of − 500 N are shown in Fig. 10.

In the structural analysis calculations for the condition of 3 MPa pressure and − 500 N load, the Von Mises stress was found to be 74.34 MPa, and the total deformation was 0.075 mm.

**Fig. 11:**
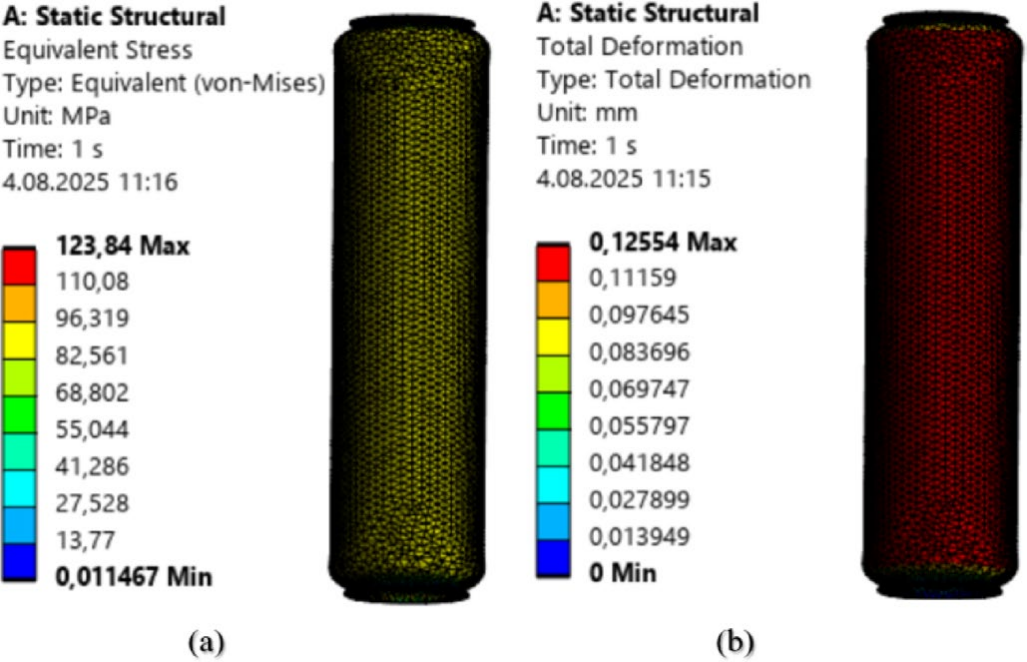
Structural Analysis (**a**) Von Mises (**b**) Total Deformation.

The structural analysis results for an input pressure of 5 MPa and a load of − 1300 N are presented in Fig. 11.

In the structural analysis calculations for the condition of 5 MPa pressure and − 1300 N load, the Von Mises stress was found to be 123.84 MPa, and the total deformation was 0.12 mm.

To better understand the relationship between the GPR algorithm model and the ANSYS structural analysis results, an error analysis was performed. The error rates can be determined using the equations given below.

7 $$\:Total\:Error\:\left(\%\right)=\frac{GPR\:\:result-Simulation\:result}{GPR\:\:result}$$

Based on this equation, an error analysis was conducted to evaluate the uncertainties between the experimental and simulation results. According to the study, the comparison of the GPR results with the ANSYS simulation results, along with a summary of the total error rate, is presented in Table 3.

**Table 3:**
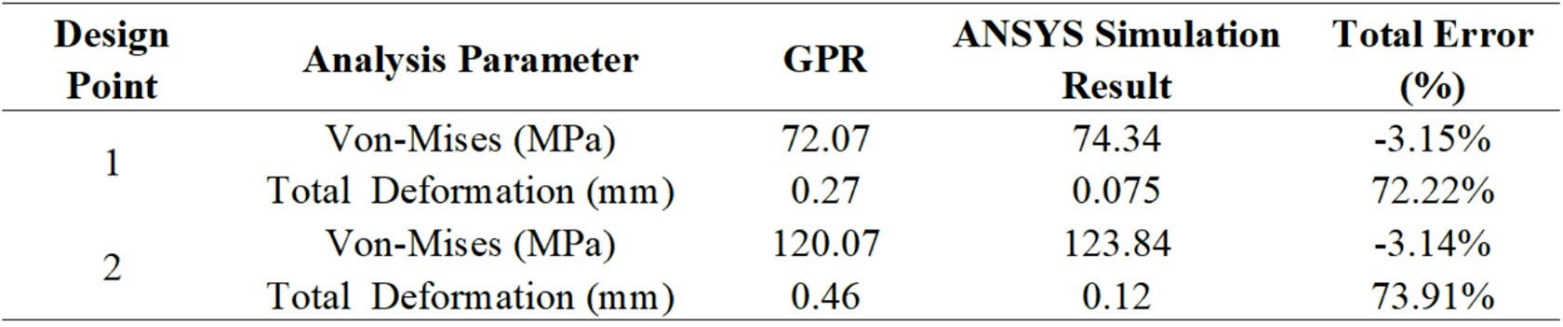
Error Analysis.

Accordingly, the prediction results obtained using the Gaussian Process Regression (GPR) method were compared with the ANSYS simulation outputs. For both analysis points, the error rate of the GPR model for Von Mises stress was below 3%, indicating a high level of predictive accuracy. However, the error rates for total deformation values were found to exceed 70%. This suggests that the GPR model is not sufficiently sensitive in predicting low-amplitude deformations. Therefore, it can be observed that, in chimney design, the GPR model produces reliable results for Von Mises stress, considered a more critical parameter than total deformation in structural analysis.

Due to the high error percentages observed in predicting the displacement target (P3) at the design points between the GPR model (ARD-SE, standardize = on) and the ANSYS structural analysis results, the applied methods were re-evaluated.

First, the total deformation output from ANSYS structural analysis, which was used for training the model and comparing the GPR results, was carefully reviewed. Since the boundary conditions remained unchanged, the primary factor influencing changes in total deformation was the mesh configuration. A convergence study was conducted until all results became independent of the mesh settings, thereby confirming the numerical reliability of the ANSYS results. Furthermore, as the total deformation value is not given at a single point but rather at any location along the entire body, its magnitude varies with different load cases and amplitudes. Consequently, predicting this value accurately is inherently challenging.

Secondly, the possibility of achieving lower error percentages in the GPR model through different settings was investigated. For P3, both z-score and log(1 + P3) transformations were tested, while the SE-ARD and Matérn-5/2 kernels were compared using Bayesian hyperparameter optimization combined with 5-fold cross-validation. Although these trials reduced the in-model absolute error to the micron level, the error rate at two independent validation points remained above 70%, similar to the initial case. The primary reasons are (i) the inflation of percentage error due to the denominator effect at very small displacement amplitudes, and (ii) a global scaling bias between the training labels and the ANSYS reference values.

In addition, for structural analyses performed using finite element software such as ANSYS, the standards specify that the maximum allowable total deformation for a 3 m-high steel chimney should be at least 3 mm (0.1% of the chimney height)^39^. Because the deformation is under 3 mm and von Mises stress predictions are accurate, these error percentages are acceptable for this design.

Below are shown both the length scales in standardized space and their corresponding values ​​converted to raw physical units (transformation: $\:{\mathcal{l}}_{\text{d}}^{\text{r}\text{a}\text{w}}={\mathcal{l}}_{\text{d}}^{\text{s}\text{t}\text{d}}\cdot\:\text{s}\text{t}\text{d}\left({\text{X}}_{\text{d}}\right)$).

P3 - Total Deformation (mm).

- Kernel: Squared Exponential (ARD).
- Length scales (std): $\:{\mathcal{l}}_{\text{P}1}=3.04268,{\mathcal{l}}_{\text{P}2}=3.02382$
- Length scales (raw): $\:{\mathcal{l}}_{\text{P}1}^{\text{r}\text{a}\text{w}}=4.04271\text{M}\text{P}\text{a},{\mathcal{l}}_{\text{P}2}^{\text{r}\text{a}\text{w}}=894.907\text{}\text{N}$
- Signal variance: $$\:{{\upsigma\:}}_{\text{f}}^{2}=2.57537\times\:{10}^{-14}{\text{}\text{m}\text{m}}^{2}$$
- Noise variance (for information): $\:{{\upsigma\:}}_{\text{n}}^{2}=1.51524\times\:{10}^{-6}{\text{}\text{m}\text{m}}^{2}$

P4 - Equivalent (von Mises) Stress (MPa).

- Kernel: Squared Exponential (ARD).
- Length scales (std): $\:{\mathcal{l}}_{\text{P}1}=0.112238,{\mathcal{l}}_{\text{P}2}=0.0919009$
- Length scales (raw): $\:{\mathcal{l}}_{\text{P}1}^{\text{r}\text{a}\text{w}}=0.149127\text{M}\text{P}\text{a},{\mathcal{l}}_{\text{P}2}^{\text{r}\text{a}\text{w}}=27.1983\text{}\text{N}$
- Signal variance: $$\:{{\upsigma\:}}_{\text{f}}^{2}=4.17694\times\:{10}^{-9}{\text{M}\text{P}\text{a}}^{2}$$
- Noise variance (for information): $\:{{\upsigma\:}}_{\text{n}}^{2}=0.107867{\text{M}\text{P}\text{a}}^{2}$

The training protocol involved model selection through 5-fold cross-validation with a random seed of 42. Alternative kernels, including Matérn-5/2, as well as target scaling (z-score and log(1 + P3)), were tested. The final selection was made for SE-ARD using the hyperparameters specified above.

## Conclusion

In this study, a chimney with a diameter of Ø500 mm, made from 2 mm thick AISI 316 stainless steel, was designed and comprehensively examined. Structural analyses were first performed on the entire chimney system and then separately on the 3-meter-long intermediate modules that constitute the chimney. In this study, SolidWorks Simulation was utilized for a comprehensive analysis of the entire chimney under its own weight, with a focus on overall stress and deformation at the supports. Meanwhile, ANSYS Workbench was employed for a detailed module-level analysis under combined loads, providing parametric datasets for the GPR model. The complementary use of both tools ensured holistic system evaluation, refined module insights, and cross-verification of results, thereby improving the reliability of the structural analysis.

Following confirmation that the structural analysis results were consistent with the literature, multiple structural parametric stress analyses were conducted on the intermediate modules under both force and static pressure. Subsequently, the Gaussian Process Regression machine learning model was applied to these analyses to predict values outside the original dataset. The investigation involved both field testing and finite element studies.

For the entire chimney system, a finite element analysis was conducted in Solidworks Simulation (academic version) by considering a total load of 22,000 N resulting from the system’s self-weight.

In this analysis, the final mesh consisted of 63,218 elements and 120,729 nodes, with an average element size of 74.5 mm. The chimney body and supporting components were modelled as solid meshes, with tetrahedral discretisation. A mesh convergence study was carried out to ensure accuracy. In Solidworks Simulation, structural analysis was conducted with the FFEPlus iterative solver, and the solution was iterated until mesh-independent results were obtained.

According to the findings, the maximum stress occurred at the chimney clamps, with a value of 28 MPa. Based on this value, the safety factor was calculated as 8.39. The total deformation was determined to be 0.58 mm, which is within acceptable limits for the chimney design.

Secondly, structural analyses were conducted for the 3-meter-long intermediate modules that comprise the chimney. These analyses were performed in the ANSYS Workbench academic structural analysis module. For each intermediate module, a force of 1,000 N corresponding to its share of the total chimney weight was applied, along with an internal pressure of 5 MPa and gravitational acceleration. This allowed for the evaluation of the module’s behavior under its own weight, combined with the applied pressure. Structural analysis was conducted with the Sparse Direct Solver. The model was refined to 32,237 elements and 21,774 nodes, with an average element size of 10 mm. The minimum element quality was 0.09, and the maximum was 0.97. Tetrahedral discretization was applied to the mesh structure. Based on this mesh, the maximum stress was found to be 119.84 MPa, corresponding to a safety factor of 1.71. The maximum total deformation was 0.46 mm, which is consistent with values reported in the literature.

To verify the accuracy of the structural analyses, a test setup was prepared in accordance with the EN 1859 standard, and pressure and leakage tests were conducted on the chimney modules. No deformation was observed during these tests.

Following this, the validated structural analysis module was used to perform the parametric analysis, providing the dataset for the Gaussian Process Regression (GPR) model, which enabled further predictive modelling.

To generate inputs for the GPR model and enable prediction capability, 50 datasets were created using the parametric analysis feature of the ANSYS Workbench academic structural analysis module. The dataset consisted of applied pressure (P1) and Z-directional force (P2) as input variables, and total deformation (P3) and equivalent (Von Mises) stress (P4) as target variables. The model was trained in MATLAB and subsequently executed.

For two input combinations not included in the initial dataset^3,5^, (MPa) and [− 500, −1300] (N), the GPR algorithm was used to make predictions. For the first case, with an input of 3 MPa pressure and − 500 N load, the predicted Von Mises stress was 72.07 MPa, and the total deformation was 0.27 mm. For the second case, with 5 MPa pressure and − 1300 N load, the predicted Von Mises stress was 120.07 MPa, and the total deformation was 0.46 mm. Based on these results, the GPR model achieved extremely high accuracy in predicting both deformation and stress values, with an R² value exceeding 0.999, consistent with the existing literature.

For the same input points, structural analysis calculations yielded the following results: for 3 MPa pressure and − 500 N load, a Von Mises stress of 74.34 MPa and a total deformation of 0.075 mm; and for 5 MPa pressure and − 1300 N load, a Von Mises stress of 123.84 MPa and a total deformation of 0.12 mm.

A comparison between the prediction results obtained from the Gaussian Process Regression (GPR) method and the ANSYS simulation outputs showed that, for both analysis points, the error rate for Von Mises stress was below 3%, indicating high predictive accuracy. However, the error rates for total deformation exceeded 70%, suggesting that the GPR model was not sufficiently sensitive in predicting low-amplitude deformations. Therefore, it can be concluded that, in chimney design, the GPR model produces reliable results for Von Mises stress, which is a more critical parameter than total deformation in structural analysis. The high error percentages in predicting total deformation (P3) using the GPR model primarily result from the intrinsic difficulty of modeling displacements that vary across the entire structure and remain very small in magnitude. Although mesh convergence studies confirmed the numerical reliability of ANSYS results and various transformations/kernels were tested, the percentage error at independent validation points exceeded 70% due to denominator effects and scaling bias. Nevertheless, von Mises stress, the most critical structural parameter, was accurately predicted by the model. Since the total deformation remained well below the allowable 3 mm limit for a 3 m-high steel chimney, the observed error percentages can be regarded as acceptable within the design context.

Many chimney sales/design companies, to shorten the bidding process, rely on rule-based approaches derived from experience rather than conducting a full-scale finite element analysis (FEA) for each project variant. However, once a bid is awarded and a detailed FEA is performed, this practice often reveals solutions that are either overly conservative (and thus costly) or insufficiently safe (and therefore risky), ultimately leading to unnecessary costs being borne by either the supplier or the client.

In this study, the proposed integrated FEA–ML approach addresses this gap by generating instant predictions under new boundary conditions through a surrogate model trained on a limited set of converged FEA runs. Even for the simplest chimney problem, results can be obtained in approximately 2 min as an alternative to the ~ 30 min required by a conventional FEA solution (yielding an average 15× time saving); in broader parameter scans, this benefit becomes even more pronounced. This enables more realistic cost estimations during the bidding stage while reducing unnecessary safety margins and mitigating safety risks.

When design is performed within the validated domain, Von Mises equivalent stress outputs can be rapidly screened. Within this framework, the proposed approach not only reduces time and CPU-hour costs but also systematically supports the development of safer and more economic solutions. Thus, the study contributes to the literature by enhancing awareness among professionals, designers, and readers working in this field.

In future studies, the suitability of different machine learning models for deformation prediction can be investigated. The parameters of the GPR model can be optimized to capture small deformation values more accurately. Additionally, the effects of corrosion and long-term material degradation can be incorporated into the scope of the analysis.

## Data Availability

The datasets used and/or analyzed during the current study are available from the corresponding author on reasonable request.
